# Early-Stage, BRCA-Associated Ovarian Cancer Detected by Papanicolaou Smear: A Case Report

**DOI:** 10.7759/cureus.40481

**Published:** 2023-06-15

**Authors:** Yun-Han Su, Hsiu-Wei Su, Chih-Ku Liu, Chien-Hsing Lu, Shih-Tien Hsu

**Affiliations:** 1 Department of Obstetrics, Gynecology & Women's Health, Taichung Veterans General Hospital, Taichung, TWN; 2 Department of Obstetrics, Gynecology & Women’s Health, Taichung Veterans General Hospital, Taichung, TWN; 3 Department of Translational Medicine, Institute of Biomedical Sciences and Rong-Hsing Research Center for Translational Medicine, National Chung-Hsing University, Taichung, TWN; 4 Center for General Education, Ling Tung University, Taichung, TWN; 5 School of Medicine, China Medical University, Taichung, TWN

**Keywords:** gynecological pathology, ovarian cancer, cancer screening, early cancer detection, abnormal pap smear

## Abstract

Historically known as a "silent killer", ovarian cancer is often diagnosed at an advanced stage. We describe an unusual case of stage I, ovarian, high-grade serous carcinoma detected by a routine Papanicolaou (PAP) smear, with no abnormal physical, imaging, or laboratory findings. A 53-year-old woman with newly diagnosed triple-negative breast cancer received a screening Pap smear, which showed malignant cells not coming from the breast or uterine cervix. Pelvic examination, cervical biopsy, and gynecologic ultrasonography found no abnormality. Endometrial curettage yielded free-floating adenocarcinoma cells. The immunohistochemical stain result indicated ovary or fallopian tube cancer. Complete cytoreductive surgery was performed, and high-grade serous carcinoma of bilateral ovaries, FIGO stage IB, was diagnosed. Although extremely rare, when malignant cells not originating from the uterine cervix are detected on a Pap smear, it may lead to an early diagnosis of ovarian cancers, and this warrants further comprehensive workup.

## Introduction

Ovarian cancer is historically called a “silent killer.” Women with ovarian cancer are often diagnosed at an advanced stage. The overall stages at the diagnosis of ovarian cancer are 23% in stage I, 8% in stage II, 34% in stage III, and 26% in stage IV [1]. There is still no efficient screening method for early ovarian cancer detection in prospective randomized controlled trials [1]. Most patients with ovarian cancer present with nonspecific symptoms. The most common symptom presenting in early-stage ovarian cancer is abdominal or pelvic pain [2]. The most common symptom in an advanced stage is abdominal swelling caused by ascites. Some women experience other non-specific symptoms, such as pelvic or abdominal pain, indigestion, and altered bowel habits, before diagnosis [1].

The Papanicolaou (Pap) smear has been widely used to screen for precancerous and malignant cervical diseases. In 1946, Papanicolaou purposed the diagnostic value of a cervicovaginal smear for its ability to collect exfoliated cancer cells from the lower genital tract [3].

The uterine cervix is rarely metastasized by ovarian cancers. First, the lymphatic drainage system directs cancer cells away from the cervix. Secondly, the cervix consists mainly of fibrous tissue, which is not favored by ovarian cancer cells to seed on [4]. Nevertheless, exfoliated ovarian cancer cells may travel through the fallopian tubes and reach the endocervix, hence they are occasionally collected by a cervicovaginal smear. The detection of ovarian cancer cells by a cervicovaginal smear usually indicates an advanced clinical stage, as reported in the literature [5-7].

Here, we present a case with early-stage, high-grade serous ovarian malignancy detected by a screening Pap smear while she received neoadjuvant chemotherapy for her breast cancer.

## Case presentation

Case presentation, physical, imaging, and laboratory findings

A 53-year-old Taiwanese woman presented with a right breast lump for more than one month. Before that, her only medical record was cervical condyloma treated by excision. She received a breast biopsy and was diagnosed with triple-negative, clinical stage Ic invasive ductal carcinoma of her right breast in a local hospital. While she was having neoadjuvant chemotherapy with Doxorubicin, Cyclophosphamide, and Bevacizumab, she received a screening Pap smear. Malignant cells, not squamous cell carcinoma or adenocarcinoma, were identified.

**Figure 1:**
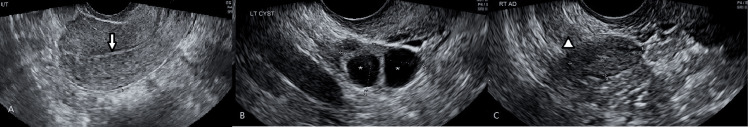
Transvaginal ultrasound of uterus and ovaries (A) the uterine body with normal endometrial lining; (B) two small cysts, measuring 1.3 cm and 1.5 cm respectively, in her left ovary, which usually indicated benign cysts; (C) normal appearance of the right ovary, measuring 2.2 cm x 1.2 cm Arrow: the endometrial lining. Asterisks: simple ovarian cysts. Arrowhead: right ovary

She was transferred to our hospital, a medical center, for further evaluation to clarify the origin of the malignant cells. Pelvic examination and gynecologic ultrasonography found no abnormality (Figure 1). Colposcopy showed no cervical anomaly. Abdominal computer tomography (CT) revealed only a 2.2 cm mass protruding from the uterus, favoring a myoma, and no other intra-abdominal lesion or lymphadenopathy. Her tumor markers, listed below, were all within normal limits: cancer antigen 125 (CA-125) 6.04 U/mL (<35 U/mL), cancer antigen 19-9 (CA 19-9) 11.00 U/mL (<37 U/mL), cancer antigen 15-3 (CA 15-3) 20.26 U/mL (<25 U/mL),; and carcinoembryonic antigen (CEA) level 1.33 ng/ml (<5 ng/ml).

Tissue biopsy evaluation

**Figure 2:**
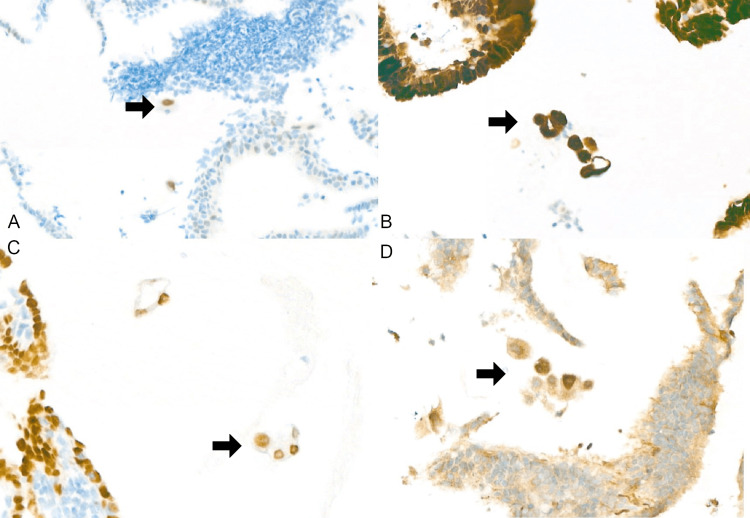
The endometrial curettage and IHC stain findings (A) IHC stain of the free-floating showed positive p53 (x200 magnification); (B) IHC stain of the free-floating cells showed positive p16 (x200 magnification); (C) IHC stain of the free-floating cells showed positive PAX8 (x200 magnification); (D) IHC stain of the free-floating cells showed positive WT-1 (x200 magnification) Arrows: adenocarcinoma cells. IHC stain: immunohistochemical stain

A random cervical biopsy revealed chronic cervicitis. Endocervical curettage found normal glandular cells. Endometrial curettage yielded proliferative endometrium with a few free-floating adenocarcinoma cells and normal endometrial glands. Therefore, the nature of the malignant cells was still undetermined. We performed immunohistochemical (IHC) stains. These tumor cells were positive for Wilms’ tumor 1 (WT1), Paired-box gene 8 (PAX8), p53, and p16, and they were negative for estrogen receptor (ER), progesterone receptor (PR), gross cystic disease fluid protein 15 (GCDFP-15), and GATA binding protein 3 (GATA3). We compared the previous IHC findings of breast specimens done at another hospital, which were negative for ER, PR, WT1, and PAX8. The IHC findings suggested that these free-floating cells, collected from the Pap smear, were more likely to be originated from the ovary or fallopian tube, rather than from metastasis of her breast cancer (Figure 2).

Surgery and pathologic findings

**Figure 3:**
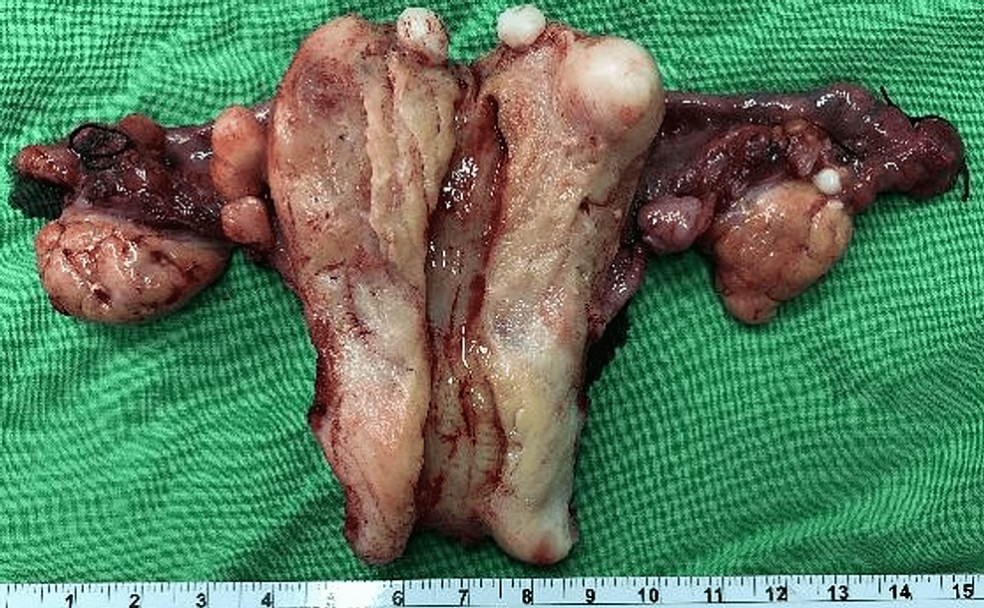
The resected specimen The specimen consists of a resected uterus, bilateral fallopian tubes, and ovaries. Grossly, there are only several myomas. No other tumor or other abnormal structure is seen in the endometrial cavity and myometrium.

The patient underwent complete cytoreductive surgery, including total hysterectomy, bilateral salpingo-oophorectomy, pelvic lymph node dissection, para-aortic dissection, and omentectomy. The uterus, bilateral adnexa, and other pelvic organs appeared normal by the surgeon’s inspection and palpation (Figure 3).

**Figure 4:**
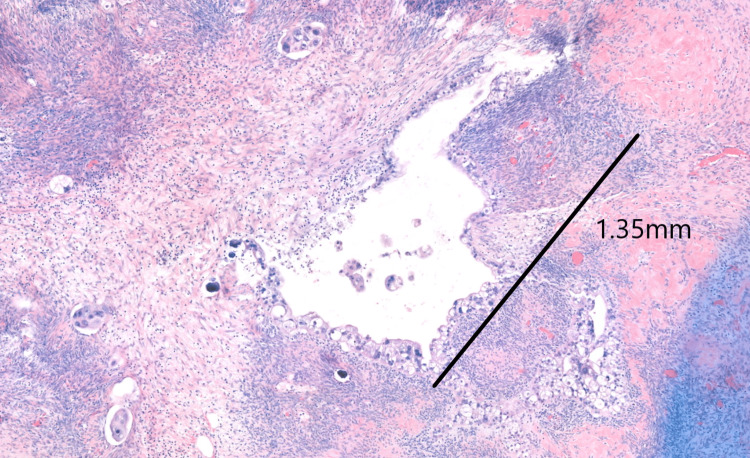
The histologic examination of the left ovary The largest tumor lesion site in the left ovary. It showed poor differentiated serous carcinoma (Hematoxylin and Eosin, x30 magnification). The size of the lesion is labeled.

**Figure 5:**
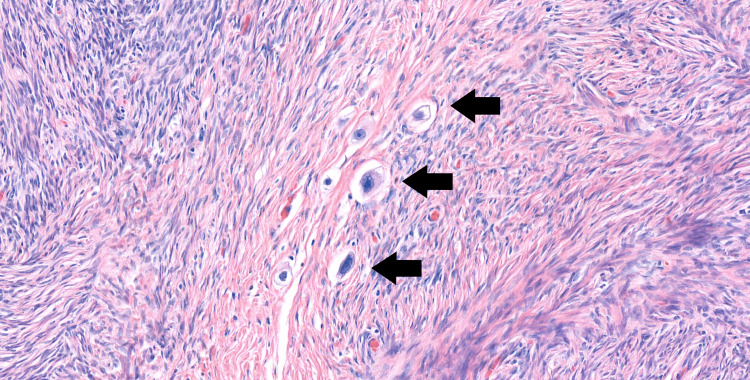
The histologic examination of the right ovary Only some single cancer cells were found in the right ovary. Arrows: carcinoma cells

The patient had no obvious ascites, and peritoneal washing cytology was negative for malignancy cells. The pathology, however, revealed high-grade serous carcinoma of bilateral ovaries (Figure 4, Figure 5).

**Figure 6:**
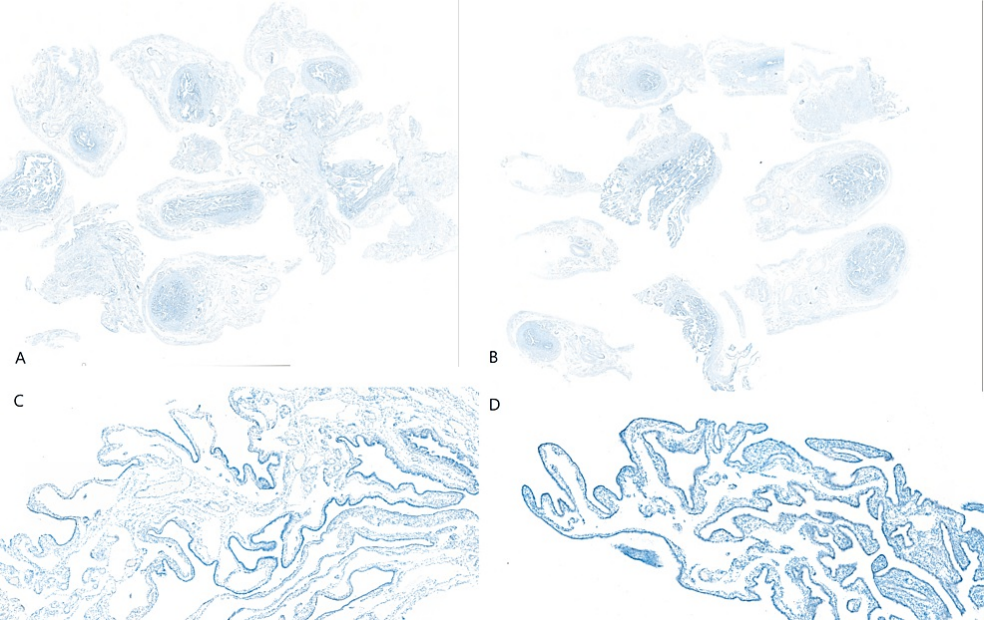
The section of bilateral fallopian tubes following SEE-FIM protocol. (A) The section of the whole left fallopian tube (p53, x2 magnification); (B) The section of the whole right fallopian tube (p53, x2 magnification); (C) Part of the left fallopian tube section, no atypical cell or malignant cell found, with the p53 wild-type pattern (p53, x20 magnification); (D) Part of the right fallopian tube section, no atypical cell or malignant cell found, with p53 wild-type pattern (p53, x100 magnification).

The endometrium was thoroughly sampled for a slide review (all for section) and no atypical cells or malignant cells were found. The bilateral fallopian tubes were sectioned following the sectioning and extensively examining the fimbriated end (SEE-FIM) protocol, and no atypical cells or malignant cells were found (Figure 6).

No masses or tumors were observed on the gross specimen of the omentum by inspection and palpation. The omentum was sectioned with one-centimeter intervals. The three most representative sections were selected for microscopic examination, and no atypical cells or malignant cells were found. The uterine serosa, myometrium, cervix, parametrium, pelvic lymph nodes, and para-aortic lymph nodes were all free of tumors. We performed WT1, PAX8, GCDFP-15, GATA-3, special AT-rich sequence-binding protein 2 (SATB2), and SRY-box transcription factor 10 (SOX10) on the specimen of ovaries. The WT1 and PAX8 were positive while the GATA-3, SATB2, and SOX10 were negative. We checked the p53 signature of bilateral fallopian tubes, and the result was the wild-type pattern. Therefore, the cancer was considered a new primary ovarian cancer. Theoretically, cancer cells could have been present in the ascites at some moment, and the stage should be IC, but they were not detected in the final peritoneal washing cytology. According to the above objective pathologic findings, the FIGO stage was IB.

Postoperative treatment and follow-up

After the surgery, the patient received four cycles of chemotherapy with Docetaxel and Carboplatin, followed by a bilateral total mastectomy. The pathologic report revealed stage I triple-negative breast cancer. The patient had regular follow-ups for both ovarian cancer and breast cancer. Till the time this article was drafted, four years after the treatment, there was no sign of any tumor recurrence.

During her follow-ups, more of her family history was obtained. Her mother and grandmother died of endometrial cancer many years ago. Her sister was also diagnosed with triple-negative breast cancer six months after the patient had finished her treatment. Due to multiple BRCA-related cancer family histories, the patient and her family members received genetic testing. The patient, her sister, and her daughter were found to have the BRCA 1 c.212 C>A mutation.

## Discussion

Epithelial ovarian cancer is one of the leading causes of death in gynecologic malignancies. In its early stage, affected women generally have no or few specific symptoms. Therefore, the disease is commonly detected at an advanced stage. Epithelial ovarian cancers are classified into five main types: high-grade serous, low-grade serous, endometrioid, clear cell, and mucinous [1]. The most prevalent histological type is high-grade serous carcinoma, which is usually diagnosed at FIGO stage III (51%) or IV (29%) [1]. Currently, there is still no reliable screening method to detect early-stage ovarian cancer. Universal screening with transvaginal ultrasound alone or in combination with CA-125 did not decrease mortality from ovarian cancer in average-risk women [8]. Major cancer societies, including the U.S. Preventative Services Task Force, the American Cancer Society, the American Congress of Obstetricians and Gynecologists, and the National Comprehensive Cancer Network (NCCN), all recommend against routine screening for all populations.

For high-risk women, especially those carrying a BRCA gene mutation, the approach is very different. The lifetime risk of ovarian cancer is 39% to 59% in BRCA 1 gene mutation carriers and 11% to 20% in BRCA 2 gene mutation carriers [9]. The NCCN suggested comprehensive counseling and considering risk-reducing salpingo-oophorectomy (RRSO), favorably between 35 and 40 years old, if childbearing is completed. For those women not receiving RRSO, despite no proven benefit, yearly transvaginal ultrasound combined with serum CA-125 testing starting from age 30 can be considered [10].

Ovarian cancer cells spread mainly through the exfoliation of cells along the peritoneal surface or lymphatic dissemination, and hematogenous metastasis is less common [8]. Daw et al. purposed that the high content of fibrous tissue in the cervix, the small size of the cervix, and the lymphatic drainage of the pelvis in a direction away from the cervix may be the reasons why the cervix is unfavorable to metastasis [4]. In our case, we suspected that in rare circumstances, ovarian tumor cells drop on fimbria, travel through the fallopian tubes and the uterine cavity, and reach the surface of the uterine cervix without seeding into any organ on the path.

After George Papanicolaou first published his work on the use and diagnostic procedure of vaginal smear for uterine cancer [11], he and his colleagues published another article to describe the diagnostic value of a cervicovaginal smear for its ability to collect exfoliated cancer cells from the lower genital tract or even other intra-pelvic organs like the urinary bladder or kidneys [3]. Later articles suggested that in a routine Pap smear, the detection of free-floating tumor cells, psammoma bodies, or atypical glandular cells (AGC) on cervical cytology may indicate malignancies other than those of cervical origin [5]. The free-floating tumor cells or other abnormal cells detected by Pap smear may come from tumor direct extension if the tumor was adjacent to the cervix; along with ascites flow if there was ascites formation or through patent fallopian tubes.

Mulvany et al. stated in a review article that there are various ways for tumor cells to spread to the cervix, and cancers of the endometrium, fallopian tubes, ovaries, or even extra genital organs all have the possibility to present with malignant cells in a Pap smear [12]. Those findings often indicate disseminated diseases. To distinguish the real origin, a thorough review of specific diagnostic features of each suspicious organ is necessary.

From our literature review, non-cervical cancers presenting with abnormal Pap smear findings were not rare. However, in almost all articles, the cancers were diagnosed at a late stage, i.e., stage III or stage IV. A six-year follow-up study reported that the positive predictive value of malignant cytology by the Pap smear for all kinds of gynecological or non-gynecological malignancies was 89.7%, with endometrial carcinoma being the most frequent diagnosis [13]. In another article on abnormal Pap smear findings in extrauterine malignancies, the ovary, fallopian tubes, and gastrointestinal tract were the three most common origins [7]. Nwanodi et al. stated in a review that 10% to 40% of ovarian cancer demonstrated malignant cells on cervicovaginal cytology [5]; nine out of 11 were at grade III or a more advanced stage. Tepeoğlu et al. reviewed six cases of ovarian serous borderline tumors presented as atypical glandular cells and psammoma bodies in a Pap smear [6], and none were diagnosed at a FIGO stage less than IIB. We summarize similar cases from the literature in Table 1. To the best of our knowledge, our case is the first reported case in which stage IB ovarian cancer was detected by a Pap smear.

**Table 1:** Ovarian cancer cases diagnosed via abnormal cervicovaginal cytology AGC: atypical glandular cell. AGUS: atypical glandular cells of undetermined significance

Authors	Cervicovaginal cytology	Diagnosis	Stage
Athavale and Chia [18]	AGC, psammoma bodies	Ovarian serous borderline malignancy	IIIC
Benson [19]	Adenocarcinoma, psammoma bodies	Ovarian serous adenocarcinoma	III
Dance et al. [20]	Poorly differentiated adenocarcinoma	Ovarian adenocarcinoma	III
Dance et al. [20]	Psammoma bodies, adenocarcinoma	Undifferentiated ovarian serous cystadenocarcinoma	III
Fox [21]	Adenocarcinoma	Ovarian papillary adenocarcinoma	III
Fox [21]	Adenocarcinoma	Ovarian adenocarcinoma	IC
Kanda [22]	Psammoma bodies, adenocarcinoma	Ovarian adenocarcinoma	IC
Kanda [22]	Psammoma bodies, adenocarcinoma	Ovarian adenocarcinoma	IIA
Kirkland et al. [23]	Adenocarcinoma, psammoma bodies	Ovarian serous adenocarcinoma	III
Mendez et al. [24]	Adenocarcinoma	Ovarian endometrioid adenocarcinoma	IIIA
Nwanodi et al. [5]	AGUS	Ovarian serous adenocarcinoma	IIIC
Nicklin et al. [25]	psammoma bodies	Ovarian serous borderline malignancy	IIB
Qizilbash [26]	Malignant glandular cells with psammoma bodies	Ovarian serous adenocarcinoma	III
Qazi et al. [27]	AGC, psammoma bodies	Ovarian papillary serous cystadenoma of borderline malignancy	IIC
Simon et al. [28]	AGUS	Ovarian serous adenocarcinoma	IIIB
Tepeoğlu [6]	AGC	Ovarian serous borderline malignancy	IIIB
Zhang and Selvaggi [29]	AGC, psammoma bodies	Ovarian serous borderline malignancy	IIIA
Su	other malignant neoplasm	Ovarian serous adenocarcinoma	IB

Whitlock et al. reported low sensitivity of Pap smears in the diagnosis of precancer or cancer lesions, mainly because of a lack of sufficient abnormal cells on the slides [14]. So, a Pap smear is not a reliable way to detect ovarian cancers with small tumor volumes. Apart from a traditional Pap smear, some other methods may have the potential in detecting ovarian cancers. Ostuka et al. stated in their study that endometrial cytology had higher sensitivity in detecting early-stage ovarian, tubal, or peritoneal high-grade serous carcinoma than cervicovaginal cytology [15]. Kinde et al. reported the detection of some commonly mutated genes associated with endometrial cancers and ovarian cancers by analyzing the fluid collected from a standard liquid-base Pap smear, with a sensitivity of 100% for endometrial cancer and 41% for ovarian cancer [16]. Other techniques, such as analyzing DNA methylation in certain genes extracted from scraped cervical cells, also showed promising results for the early detection of endometrial and ovarian cancers [17]. However, with many techniques mentioned and developed, there is still not a single screening method that can be effective enough to reduce the mortality of high-grade serous ovarian cancer.

## Conclusions

Pap smear, a widely accepted screening test for cervical cancer, has the potential to detect ovarian cancer, though most reported cases were at a later clinical stage. We present a case with early-stage ovarian cancer detected by a screening Pap smear, with an absence of any abnormal findings by physical examination, serum tumor markers, pelvic ultrasound, and abdominal CT. It reminds us that although rare, an abnormal Pap smear may lead to the early diagnosis of non-cervical cancers, and it warrants further comprehensive workup. With the development of new methodologies, the early detection of tumor cells or DNAs from ovarian cancer by this relatively simple exam seems achievable and more studies regarding the feasibility and cost-effectiveness of new techniques are necessary.

## References

[1] Torre LA, Trabert B, DeSantis CE (2018). Ovarian cancer statistics, 2018. CA Cancer J Clin.

[2] Chan JK, Tian C, Kesterson JP (2022). Symptoms of women with high-risk early-stage ovarian cancer. Obstet Gynecol.

[3] Papanicolaou GN (1946). Diagnostic value of exfoliated cells from cancerous tissues. J Am Med Assoc.

[4] Daw E (1972). Extragenital adenocarcinoma metastatic to the cervix uteri. Am J Obstet Gynecol.

[5] Nwanodi O, Choi C, Khulpateea N (2008). Cervicovaginal cytology and diagnosis of ovarian or peritoneal cancer: case report and literature review. Arch Gynecol Obstet.

[6] Tepeoğlu M, Ozen O, Ayhan A (2013). Ovarian serous borderline tumor detected by conventional papanicolaou smear: a case report. Acta Cytol.

[7] Gupta D, Balsara G (1999). Extrauterine malignancies. Role of Pap smears in diagnosis and management. Acta Cytol.

[8] Berek JS (2019). Berek & Novak’s Gynecology. https://shop.lww.com/Berek---Novak-s-Gynecology/p/9781496380333.

[9] Giannakeas V, Lim DW, Narod SA (2021). The risk of contralateral breast cancer: a SEER-based analysis. Br J Cancer.

[10] Daly MB, Pal T, Berry MP (2021). Genetic/familial high-risk assessment: breast, ovarian, and pancreatic, version 2.2021, NCCN Clinical Practice Guidelines in Oncology. J Natl Compr Canc Netw.

[11] Papanicolaou GN, Marchetti AA (1943). The use of endocervical and endometrial smears in the diagnosis of cancer and of other conditions of the uterus. Am J Obst Gynecol.

[12] Mulvany NJ, Mitchell G, Allen DG (2009). Adenocarcinoma cells in Pap smears. Pathology.

[13] Uyar DS, Eltabbakh GH, Mount SL (2003). Positive predictive value of liquid-based and conventional cervical Papanicolaou smears reported as malignant. Gynecol Oncol.

[14] Whitlock EP, Vesco KK, Eder M, Lin JS, Senger CA, Burda BU (2011). Liquid-based cytology and human papillomavirus testing to screen for cervical cancer: a systematic review for the U.S. Preventive Services Task Force. Ann Intern Med.

[15] Otsuka I, Kameda S, Hoshi K (2013). Early detection of ovarian and fallopian tube cancer by examination of cytological samples from the endometrial cavity. Br J Cancer.

[16] Kinde I, Bettegowda C, Wang Y (2013). Evaluation of DNA from the Papanicolaou test to detect ovarian and endometrial cancers. Sci Transl Med.

[17] Chang CC, Wang HC, Liao YP, Chen YC, Weng YC, Yu MH, Lai HC (2018). The feasibility of detecting endometrial and ovarian cancer using DNA methylation biomarkers in cervical scrapings. J Gynecol Oncol.

[18] Athavale RD, Chia KV (2002). Psammoma bodies on postmenopausal cervical smear--a rare sinister finding. J Obstet Gynaecol.

[19] Benson PA (1973). Psammoma bodies found in cervico-vaginal smears. Acta Cytol.

[20] Dance EF, Fullmer CD (1970). Extrauterine carcinoma cells observed in cervico-vaginal smears. Acta Cytol.

[21] Fox CH (1978). Adnexal malignancy detected by cervical cytology. Am J Obstet Gynecol.

[22] Kanda Y, Sato T, Tanaka S, Takashina T, Kudo R (1986). Two cases of ovarian cancer with psammoma bodies in the uterine cervical and endometrial smears (Article in Japanese). Journal of the Japanese Society of Clinical Cytology.

[23] Kirkland N, Hardy N (1979). Psammoma bodies found in cervicovaginal smears. A case report. Acta Cytol.

[24] Mendez LE, Majmudar B, Horowitz IR (2000). Atypical presentation of microscopically advanced ovarian carcinoma. South Med J.

[25] Nicklin JL, Perrin L, Obermair A, McConachie I, Cominos D (2001). The significance of psammoma bodies on cervical cytology smears. Gynecol Oncol.

[26] Qizilbash AH (1974). Ovarian carcinoma identified by psammoma bodies in the cervicovaginal and endometrial smears. Can Med Assoc J.

[27] Qazi FM, Geisinger KR, Barrett RJ, Hopkins MB 3rd, Holleman IL Jr (1988). Cervicovaginal psammoma bodies. The initial presentation of the ovarian borderline tumor. Arch Pathol Lab Med.

[28] Simon DA, Dimitrievich E (2003). Case report: detection of an advanced ovarian malignancy by cervical cytology. S D J Med.

[29] Zhang Y, Selvaggi SM (2003). Significance of psammoma bodies on a cervical sample from an asymptomatic woman. Diagn Cytopathol.

